# Specific loss of CatSper function is sufficient to compromise fertilizing capacity of human spermatozoa

**DOI:** 10.1093/humrep/dev243

**Published:** 2015-10-08

**Authors:** Hannah L. Williams, Steven Mansell, Wardah Alasmari, Sean G. Brown, Stuart M. Wilson, Keith A. Sutton, Melissa R. Miller, Polina V. Lishko, Christopher L.R. Barratt, Steven J. Publicover, Sarah Martins da Silva

**Affiliations:** 1 Reproductive and DevelopmentalBiology, Medical School, Ninewells Hospital, University of Dundee, Dundee DD1 9SY, UK; 2 Assisted Conception Unit, NHS Tayside, Ninewells Hospital, Dundee DD1 9SY, UK; 3 Department of Molecular and Cell Biology, University of California, Berkeley, USA; 4 Abertay University, Dundee DD11HG, UK; 5 Wolfson Research Institute, School of Medicine, Pharmacy and Health, Queen's Campus, University Of Durham, Stockton on Tees TS17 6BH, UK; 6 Department of Biosciences, University of Birmingham, Birmingham B152TG, UK

**Keywords:** CatSper, male fertility, sperm motility, ion channels, calcium stores, electrophysiology, failed fertilization, contraception, unexplained infertility, sperm dysfunction

## Abstract

**STUDY QUESTION:**

Are significant abnormalities of CatSper function present in IVF patients with normal sperm concentration and motility and if so what is their functional significance for fertilization success?

**SUMMARY ANSWER:**

Sperm with a near absence of CatSper current failed to respond to activation of CatSper by progesterone and there was fertilization failure at IVF.

**WHAT IS KNOWN ALREADY:**

In human spermatozoa, Ca^2+^ influx induced by progesterone is mediated by CatSper, a sperm-specific Ca^2+^ channel. A suboptimal Ca^2+^ influx is significantly associated with, and more prevalent in, men with abnormal semen parameters, and is associated with reduced fertilizing capacity. However, abnormalities in CatSper current can only be assessed directly using electrophysiology. There is only one report of a CatSper-deficient man who showed no progesterone potentiated CatSper current. A CatSper 2 genetic abnormality was present but there was no information on the [Ca^2+^]_i_ response to CatSper activation by progesterone. Additionally, the semen samples had indicating significant abnormalities (oligoasthenoteratozoospermia) multiple suboptimal functional responses in the spermatozoon. As such it cannot be concluded that impaired CatSper function alone causes infertility or that CatSper blockade is a potential safe target for contraception.

**STUDY DESIGN, SIZE, DURATION:**

Spermatozoa were obtained from donors and subfertile IVF patients attending a hospital assisted reproductive techniques clinic between January 2013 and December 2014. In total 134 IVF patients, 28 normozoospermic donors and 10 patients recalled due to a history of failed/low fertilization at IVF took part in the study.

**PARTICIPANTS/MATERIALS, SETTING, METHODS:**

Samples were primarily screened using the Ca^2+^ influx induced by progesterone and, if cell number was sufficient, samples were also assessed by hyperactivation and penetration into viscous media. A defective Ca^2+^ response to progesterone was defined using the 99% confidence interval from the distribution of response amplitudes in normozoospermic donors. Samples showing a defective Ca^2+^ response were further examined in order to characterize the potential CatSper abnormalities. In men where there was a consistent and robust failure of calcium signalling, a direct assessment of CatSper function was performed using electrophysiology (patch clamping), and a blood sample was obtained for genetic analysis.

**MAIN RESULTS AND THE ROLE OF CHANCE:**

A total of 101/102 (99%) IVF patients and 22/23 (96%) donors exhibited a normal Ca^2+^ response. The mean (±SD) normalized peak response did not differ between donors and IVF patients (2.57 ± 0.68 [*n* = 34 ejaculates from 23 different donors] versus 2.66 ± 0.68 [*n* = 102 IVF patients], *P* = 0.63). In recall patients, 9/10 (90%) showed a normal Ca^2+^ response. Three men were initially identified with a defective Ca^2+^ influx. However, only one (Patient 1) had a defective response in repeat semen samples. Electrophysiology experiments on sperm from Patient 1 showed a near absence of CatSper current and exon screening demonstrated no mutations in the coding regions of the CatSper complex. There was no increase in penetration of viscous media when the spermatozoa were stimulated with progesterone and importantly there was failed fertilization at IVF.

**LIMITATIONS, REASONS FOR CAUTION:**

A key limitation relates to working with a specific functional parameter (Ca^2+^ influx induced by progesterone) in fresh sperm samples from donors and patients that have limited viability. Therefore, for practical, technical and logistical reasons, some men (∼22% of IVF patients) could not be screened. As such the incidence of significant Ca^2+^ abnormalities induced by progesterone may be higher than the ∼1% observed here. Additionally, we used a strict definition of a defective Ca^2+^ influx such that only substantial abnormalities were selected for further study. Furthermore, electrophysiology was only performed on one patient with a robust and repeatable defective calcium response. This man had negligible CatSper current but more subtle abnormalities (e.g. currents present but significantly smaller) may have been present in men with either normal or below normal Ca^2+^ influx.

**WIDER IMPLICATIONS OF THE FINDINGS:**

These data add significantly to the understanding of the role of CatSper in human sperm function and its impact on male fertility. Remarkably, these findings provide the first direct evidence that CatSper is a suitable and specific target for human male contraception.

**STUDY FUNDING/COMPETING INTEREST(S):**

Initial funding was from NHS Tayside, Infertility Research Trust, TENOVUS, Chief Scientist Office NRS Fellowship, the Wellcome Trust, University of Abertay. The majority of the data were obtained using funding from a MRC project grant (# 4190). The authors declare that there is no conflict of interest.

**TRIAL REGISTRATION NUMBER:**

Not applicable.

## Introduction

Whilst sperm dysfunction is the single most common cause of infertility (Hull *et al.*, 1985; Irvine, 1998), currently there is no drug a man can take or that can be added to his sperm *in vitro* to improve fertility (Tardif *et al.*, 2014). A fundamental problem with developing new therapies has been the limited understanding of the physiological workings of the normal and dysfunctional spermatozoon (Barratt, 2010; Aitken and Henkel, 2011; Barratt *et al.*, 2011; Aitken and Nixon, 2013).

However, during the last 8 years major progress in our understanding has been achieved through application of the patch clamp technique (Kirichok *et al.*, 2006). This has included the characterization of the proton (H_V_1) and chloride channels (Lishko *et al.*, 2010; Orta *et al.*, 2012) and the identification and characterization of the potassium conductance underlying the regulation of sperm membrane potential (Santi *et al.*, 2010; Mannowetz *et al.*, 2013; Brenker *et al.*, 2014; Mansell *et al.*, 2014). Ca^2+^ signalling is particularly important in the regulation of sperm function (Publicover *et al.*, 2007) and potentially the most significant outcome has been the characterization of the sperm-specific Ca^2+^ channel CatSper (Ren *et al.*, 2001). CatSper-null sperm show no detectable Ca^2+^ current, defective calcium signalling, ATP depletion and abnormalities in motility (including hyperactivation; HA). CatSper knockout mice are consequently infertile (Carlson *et al.*, 2003; Jin *et al.*, 2007).

In humans, the importance of CatSper was emphasized by the demonstration that the rapid influx of calcium into the spermatozoon upon interaction with progesterone (and other substances such as prostaglandins) is via CatSper (Lishko *et al.*, 2011; Strünker *et al.*, 2011; Brenker *et al.*, 2012). Patch clamp studies have revealed that CatSper is weakly voltage sensitive and progesterone strongly potentiates CatSper currents while shifting the channel half activation voltage to a less depolarized potential (Lishko *et al.*, 2011; Strünker *et al.*, 2011). This progesterone induced, CatSper-mediated Ca^2+^ influx stimulates the acrosome reaction, either directly and/or by ‘priming’ the cell and also regulates motility (Alasmari *et al.*, 2013b; Tamburrino *et al.*, 2014). Several clinical studies have shown that suboptimal [Ca^2+^]_i_ signalling in response to progesterone is associated with oligoasthenoteratozoospermia, and with reduced fertilization at IVF (Falsetti *et al.*, 1993; Shimizu *et al.*, 1993; Oehninger *et al.*, 1994; Krausz *et al.*, 1995). The occurrence of poor [Ca^2+^]_i_ responses to agonists of CatSper and to mobilization of stored Ca^2+^ is related to IVF fertilization rates (Alasmari *et al.*, 2013a). Release of stored Ca^2+^ is more potent than CatSper activation in inducing HA and secondary release of stored Ca^2+^, downstream of CatSper activation, is likely to be required for HA (Alasmari *et al.*, 2013a). In summary, the clinical data indicate that defects in [Ca^2+^]_i_ signalling do occur and that these affect IVF fertilizing capacity.

Significant functional differences between the spermatozoa of mice and men, including regulation of motility by Ca^2+^-signalling (Alasmari *et al.*, 2013b), are such that findings cannot simply be applied to humans. Equivalent human studies rely upon the identification of ‘natural knockouts’, subfertile men with specific lesions of key genes. However, case reports of CatSper genetic abnormalities are rare. Recently, Smith *et al.* (2013) showed that sperm from a CatSper 2 deficient man had no progesterone potentiated CatSper current; consistent with the idea that CatSper is the primary channel responsible for Ca^2+^ influx. However, there was no information on the Ca^2+^ influx induced by progesterone. Further, the semen samples in this study had multiple abnormalities suggesting defects at additional loci and multiple suboptimal functional responses in the spermatozoon (Avidan *et al.*, 2003; Zhang *et al.*, 2007; Avenarius *et al.*, 2009; Bhilawadikar *et al.*, 2013). Though important, these studies do not establish either that the specific cause of infertility in these men was failure of CatSper or that inactivation of CatSper is sufficient to prevent/reduce fertilization in humans. Furthermore, they raise the possibility that loss of CatSper function affects spermatogenesis in humans, which would greatly reduce the potential suitability of CatSper as a male contraceptive target (Ren *et al.*, 2001).

Therefore, the primary objective of this study was to examine whether significant abnormalities of CatSper function can be identified in IVF patients and assess their functional significance.

## Materials and Methods

### Experimental design

Abnormalities in CatSper current can only directly be assessed using electrophysiology; however, this is a low-throughput technique. Therefore, to identify men with potential CatSper abnormalities for electrophysiology, we devised a combination of screening methods to examine calcium signalling. The primary assay was the Ca^2+^ influx induced by progesterone followed by penetration into viscous media and HA (Alasmari *et al.*, 2013a,b). In total 134 IVF patients, 28 donors and 10 patients recalled due to a history of low/failed fertilization at IVF took part in the study. The focus was examination of Ca^2+^ influx but samples were allocated to the other assays if cell number was sufficient. Samples showing a defective Ca^2+^ influx response were further examined in order to characterize the potential CatSper abnormalities. Direct assessment of CatSper was performed using electrophysiology where a consistent and robust failure of calcium signalling (i.e. repeatable in more than one semen sample) was found (Supplementary Fig. S1).

### Patient and donor numbers

Of the 134 IVF patients, 102 were screened for calcium response to progesterone, 66 for 4-aminopyridine (4-AP)-induced HA and 16 for penetration of viscous medium. A total of 111 of these patients fertilized at IVF (average 78.6 ± 22% (1 SD)), while 9 patients had total failed fertilization, 8 had less than 4 oocytes retrieved and for 6 there were no fertilization data available.

Of the 28 donors, 23 provided one or more samples (34 ejaculates in total) for the Ca^2+^ response to progesterone, 35 ejaculates for the 4-AP-induced HA assay and 18 ejaculates for the viscous media penetration test.

All of the 10 recall patients were screened for Ca^2+^ response to progesterone and one was screened for 4-AP-induced HA and viscous medium penetration.

### Ethical approval and subjects

Written informed consent was obtained from three groups: (i) patients (subfertile men who underwent assisted reproduction techniques in Ninewells Hospital, Dundee, Scotland); (ii) sperm donors (selected with no known fertility problems and normal semen parameters) (Cooper *et al.*, 2010); (iii) patients with a history of failed/low fertilization at IVF. Men were recruited in accordance with the Human Fertilisation and Embryology Authority Code of Practice (version 8) under local ethical approval (13/ES/0091) from the Tayside Committee of Medical Research Ethics B.

### Semen samples

Samples from donors and patients were used as previously described (Tardif *et al.*, 2014). Patients were selected for IVF according to clinical indications and semen quality: e.g. normal sperm concentration and motility (Cooper *et al.*, 2010) and ∼ 1 × 10^6^ progressively motile cells post-preparation. The surplus of the clinical sample used in the IVF treatment process was assessed for intracellular Ca^2+^ and where possible HA and penetration into viscous media.

### Media and preparation

Commercially available Quinn's Advantage Fertilization Medium (HTF) plus 5% human serum albumin (HSA) and Quinn's Sperm Washing Medium (SW) were used to prepare and capacitate sperm. Non-capacitating HEPES-buffered media (NCM) lacking in both albumin and bicarbonate (4.69 mM KCl, 2.04 mM CaCl_2_, 0.2 mM MgSO_4_, 97.8 mM NaCl, 2.78 mM D-Glucose, 0.33 mM Na pyruvate, 21.4 mM lactic acid and 21 mM HEPES, ∼280 mOsm/kg H_2_O, adjusted to pH 7.4 using 10 M NaOH) was used for sample re-suspension in the FLUOstar.

Spermatozoa were isolated by density gradient centrifugation (DGC) using Percoll/PureSperm diluted with SW for donors/patients respectively. After centrifugation (300*g*, 20 min), the supernatant was discarded and pellet was washed (500*g* for 10 min), resuspended in HTF, and incubated for a minimum of 2 h at 37°C in 5% CO_2_.

### Agonists and reagents

Ca^2+^ signalling was manipulated using progesterone (Lishko *et al.*, 2011; Alasmari *et al.*, 2013a) and 4-AP) (Bedu-Addo *et al.*, 2008). Reagents were purchased from Sigma Aldrich unless otherwise stated. 4-AP and progesterone were dissolved in distilled water and ethanol, respectively. Fura-2 acetoxymethyl ester (Fura-2/AM) (Molecular Probes, Invitrogen, Oregon, USA) was dissolved in dimethylsulphoxide (final concentration of 1 µM).

### Measurement of intracellular Ca^2+^

Approximately 4 million cells were prepared and assessed as previously described using a FLUOstar microplate reader (BMG Labtech Offenburg, Germany) (Alasmari *et al.*, 2013a) with minor modifications encompassing use of 0.05% Pluronic acid F127 and manganese chloride (9.1 mM). Progesterone-induced increments in the ratio of emission intensities (at 340 and 380 excitation) were used to quantify changes in [Ca^2+^]_i_ concentration (Alasmari *et al.*, 2013a,b). After adjustment for background fluorescence normalization was achieved by dividing each ratio value at time point × (Rx) by the mean fluorescence ratio value taken from hundreds of basal fluorescence ratio readings (Rbasal).

### Assessment of HA and motility

A Hamilton Thorne CEROS computer aided sperm analysis (CASA) machine (Beverley, MA, USA) was used to assess motility for semen and prepared sperm samples (as described in Supplementary Table SI) (Alasmari *et al.*, 2013a,b). Sperm were treated with 4-AP at a final concentration of 2 mM, and the percentage of hyperactivated cells was assessed (Mortimer *et al.*, 1998).

### Penetration into viscous media

This was performed as previously described (Ivic *et al.*, 2002; Alasmari *et al.*, 2013b). Methylcellulose solution was created using a 1% w/v methylcellulose in HTF media supplemented with 5% HSA. Methylcellulose solution was introduced into capillary tubes by capillary action (Vitrotubes™, Rectangle Capillaries 0.4 mm × 400 mm, VitroCom, New Jersey, USA). Prepared sperm were adjusted to ∼10–20 × 10^−6^/ml before 3.6 µM progesterone/vehicle were added to the aliquots. Following 1 h incubation at 37°C and 5% CO_2_, cell numbers at 1 and 2 cm were normalized to values from parallel, untreated controls (C/C, V/C and P/C, where C = number of cells under control conditions, V = under vehicle controls and P = after treatment with progesterone).

### Definition of failed HA and Ca^2+^ responses among donors and sub-fertile patients

A failed HA response was recorded when agonist stimulation did not induce a significant change in the percentage of hyperactivated cells compared with control (basal) level (assessed by paired two-tailed Student's *t*-test, *P* < 0.05). To define a defective Ca^2+^ response to progesterone, the 99% confidence interval was determined from the distribution of response amplitudes in 34 ejaculates from 23 normozoospermic donors: <0.41 and >4.85 delta response upon addition of progesterone.

### Fertilization rate at IVF

Oocytes were considered normally fertilized when two pronuclei (2PN) and two polar bodies were observed. In IVF, the fertilization rate was calculated from the number of oocytes normally fertilized divided by the total number of inseminated oocytes. The fertilization rate was calculated where four or more mature oocytes (metaphase II) were present. Low fertilization was defined where <30% of four or more metaphase II oocytes were normally fertilized.

### Sperm preparation for electrophysiology

Sperm were prepared, for electrophysiology using a swim-up procedure into HTF as previously described (Lishko *et al.*, 2013; Mansell *et al.*, 2014). The cells were re-suspended in capacitating media and maintained at 37°C for 4 h (5% CO_2_). Capacitated cells were transferred into petri dishes at room temperature containing standard bath solution in order to allow cells to adhere to glass coverslips. Coverslips were transferred into a perfusion chamber and perfused with standard bath solution until whole-cell configuration was achieved. The biophysical properties of individual sperm were recorded under whole-cell conditions (Lishko *et al.*, 2011; Mansell *et al.*, 2014) using borosilicate glass pipettes (10–15 MOhms) filled with Cs^+^-based divalent free solution. Gigaohm seals were formed on cytoplasmic droplets or on the midpiece region. Break in was achieved via light suction and 1 ms voltage pulses (450–610 mV). Flagellar beating was observed in all cells selected. In order to record CatSper, currents were recorded in Cs^+^-based divalent free extracellular solution evoked by a depolarizing ramp protocol imposed (−80 to 80 mV) over 2 s and membrane potential (Vm) was held at 0 mV between test pulses.

### Experimental solutions

All concentrations are in mM. Synthetic human tubular fluid (HTF): NaCl, 97.8; KCl, 4.69; MgSO_4_; 0.2; CaCl_2_, 2.04; HEPES, 21; Glucose, 2.78; Na-lactate 21.4; Na-pyruvate, 0.33; pH adjusted to 7.4 with NaOH. Capacitating medium: NaCl, 135; KCl, 5, MgSO_4_, CaCl_2_, 2; HEPES, 20; Glucose, 5; Lactic acid, 10; Na-Pyruvate, 1; NaHCO_3_, 25; fetal bovine serum, 20%; pH adjusted to 7.4 with NaOH. Standard bath solution: NaCl, 135; KCl, 5; CaCl_2_, 2; MgSO_4_,1; HEPES, 20; Glucose, 5; Na Pyruvate, 1; Na lactate, 10; pH adjusted to 7.4 with NaOH which brought [Na] to 154 mM. Non-selective cation currents flowing via spermatozoon cation channels (CatSper) were quantified using pipette (Cs-methanesulphonate, 130; HEPES, 40; Tris–HCl, 1; EGTA, 3; EDTA, 2 mM, pH adjusted to 7.4 with CsOH) and bath (Cs-methanesulphonate, 140; HEPES, 40; EGTA, 3; pH adjusted to 7.4 with CsOH) solutions devoid of Ca^2+^ and Mg^2+^ that contained Cs^+^ as the principal cation.

### Genetic screening for mutations in CatSper Complex loci

Blood samples were obtained from Patient 1 and processed for Exome sequencing (Centrillion Biosciences, Palo Alto, USA). Samples were selected using Agilent SureSelect Human all Exon v5-51Mb kit and yielded 1.83 Gbase, giving a raw coverage of 36X. Coverage for the CatSper Complex loci (CatSper 1-4, CatSperB, CatSperG and CatSperD) was verified. Sequences were mapped to human genome and sequences overlapping the loci of interest (Supplementary Table SII) were extracted and realigned using Geneious version 8.1.4 (http://www.geneious.com) and polymorphisms identified and manually verified.

### Statistical analysis

Normality of data was assessed according to the Shapiro–Wilk test. Statistical comparisons for the viscous penetration test and induced HA were made using paired two-tailed Student's *t*-tests or the analysis of variance if the data were either originally normally distributed or normalized after transformation by arcsin or square root. To define a cut-off value for a failed Ca^2+^ response, the data were log transformed and cut-off values were calculated based on mean and SD (Alasmari *et al.*, 2013a). Unpaired *t*-tests were performed on currents obtained from whole-cell patch clamp. *P* < 0.05 was considered significant. The statistical package Prism GraphPad (La Jolla, CA, USA) was used.

## Results

In total, 134 IVF patients, 28 donors and 10 patients recalled for assessment due to failed/low fertilization at IVF were included in the study. Patients and donors were screened using one or more of the three techniques depending on sample quality: Ca^2+^ response, HA and the viscous media assay.

### Screening of IVF patients and donors for Ca^2+^ influx induced by progesterone

**Figure 1:**
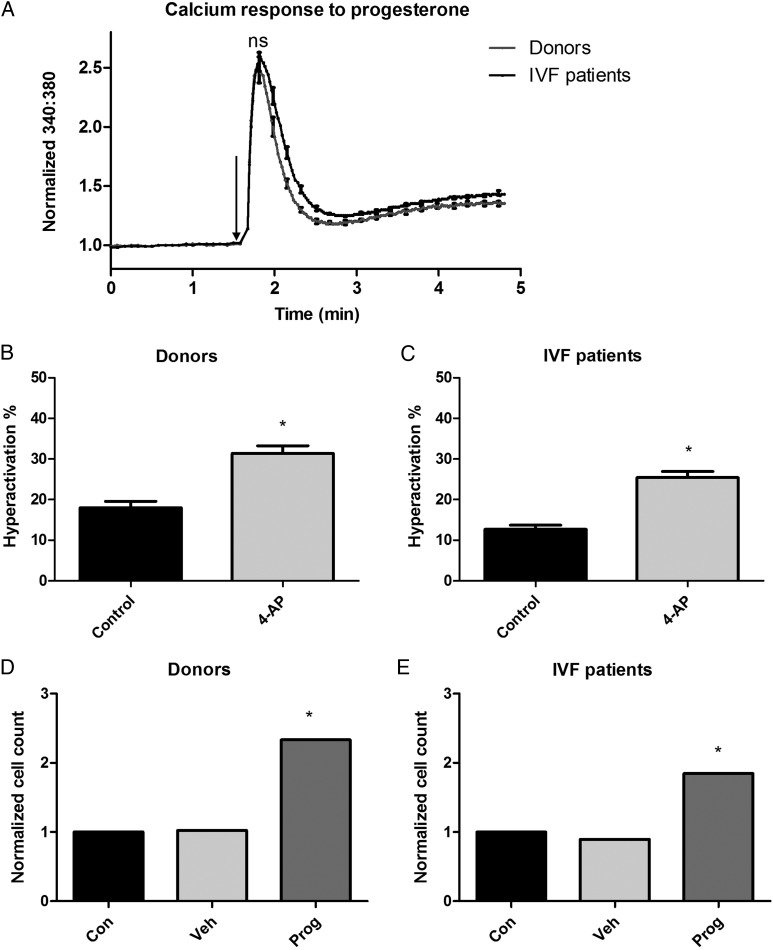
Calcium response to progesterone, hyperactivation in response to 4-AP and penetration into viscous media for spermatozoa from donors and IVF patients. (**A**) Peak calcium response to progesterone (progesterone addition indicated by the arrow) was not significantly different between donors and IVF patients (donor *n* = 34 ejaculates from 23 different donors, IVF *n* = 102), however, the plateau phase response (mean of the last 12 s of recording) was (donor versus IVF peak calcium response to progesterone, *P* = 0.003, Student's unpaired *t*-test). (**B** and **C**) 4-aminopyridine (4-AP) significantly increased hyperactivation (HA) in spermatozoa from both donors and IVF patients (*P* < 0.001 versus control, Student's paired *t*-test). HA assay donor *n* = 35, IVF *n* = 66. (**D** and **E**) Penetration into viscous media was higher after stimulation with progesterone for both donors and IVF patients (progesterone versus control, donor *n* = 18, *P* < 0.05; IVF *n* = 16, *P* < 0.05, one-way analysis of variance). Asterisk denotes statistical significance at the *P* < 0.05 level.

The majority of IVF patients (101/102; 99%) and donors (22/23; 95.7%) exhibited a biphasic [Ca^2+^]_i_ response as previously described (Alasmari *et al*, 2013a). The [Ca^2+^]_i_ transient amplitude was similar in cells from donors and patients attending for IVF treatment (Fig. 1A; donors = 2.57 ± 0.68 [*n* = 34 ejaculates from 23 different donors], IVF patients = 2.66 ± 0.68 [*n* = 102 patients], *P* = 0.63) but the plateau phase of the [Ca^2+^]_i_ response was greater in IVF patients (*P* = 0.003). In total, 9/10 patients with previous low/failed fertilization at IVF showed a normal Ca^2+^ response.

### Screening of IVF patients for HA

IVF patient samples had significantly lower basal HA than donors (Fig. 1B and C, *P* < 0.05, IVF *n* = 66, donor *n* = 35). Treatment with 2 mM 4-AP robustly and significantly increased HA for both IVF patients and donors (*P* < 0.001).

### Screening of IVF patients in the viscous media assay

Sperm from donors and IVF patients (15/18 donors [83%], 13/16 IVF patients [81%]) responded to progesterone with an increase in cell numbers at 1 cm (*P* < 0.05, Fig. 1D and E).

### Identification of defective Ca^2+^ influx induced by progesterone

Three individuals had spermatozoa that were unable to respond to progesterone. One was from the group of 10 patients recalled due to a previous low/failed fertilization at IVF (Patient 1), one was a patient undergoing IVF treatment (Patient 2) and one was a donor (Donor 1). In each case, diagnostic semen analysis was normal (Supplementary Table SI).

### Patient 1

Patient 1 had one previous cycle of IVF that resulted in a total fertilization failure for all nine oocytes retrieved. Consequently he was recalled for further analysis and provided a sample for research. He had a subsequent cycle of ICSI where 5/6 oocytes fertilized (83.3% fertilization rate). Female age was 39 years with an anti-Mullerian hormone (AMH) of 10 pmol/l. Semen analysis was normal (concentration of 66 × 10^6^/ml and motility of 60%, Supplementary Table SI).

**Figure 2:**
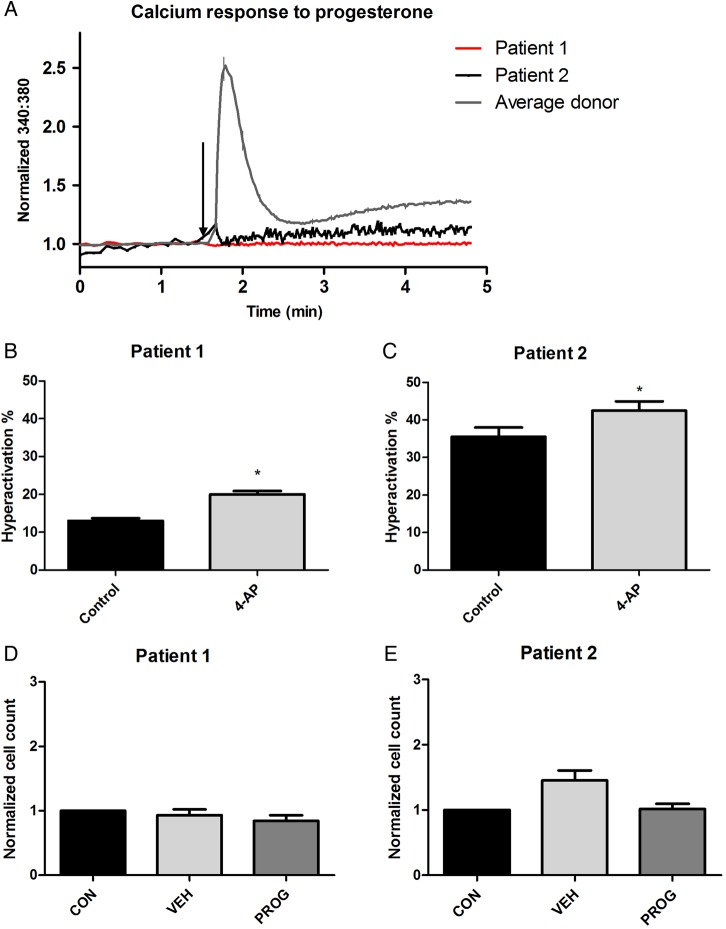
Calcium response to progesterone, hyperactivation in response to 4-AP and penetration into viscous media for spermatozoa from Patients 1 and 2. (**A**) The spermatozoa from Patient 1 and 2 showed no significant calcium response to progesterone (compared with representative average donor trace, as seen in Fig. 1A). Patient 1 trace shows an average of four different aliquots from two ejaculates separated by 7 weeks; Patient 2 trace shows analysis of one sample on the day of IVF treatment. (**B** and **C**) 4-AP induced an increase in HA in the spermatozoa from Patient 1 (*P* < 0.001) and Patient 2 (4-AP versus control, *P* = 0.01, Student's paired *t*-test). (**D** and **E**) Spermatozoa from Patients 1 and 2 showed no significant enhancement in penetration into viscous media when stimulated with progesterone.

In the first sample, the spermatozoa were unable to produce a Ca^2+^ influx induced by progesterone (Fig. 2A), though donor sperm tested at the same time and under identical conditions (positive control) gave a normal biphasic response. Furthermore, there was an abnormal response to progesterone in the viscous media assay (Fig. 2D). Stimulation of capacitated cells with 4-AP induced a significant increase in HA (*P* < 0.001, Fig. 2B).

Another sample submitted 7 weeks later showed the same result (Fig. 2A shows the average trace from four assays, two aliquots from the initial sample and two from second) and thus the cells were studied using patch clamp electrophysiology (see below).

### Patient 2

On the day of IVF treatment, prepared spermatozoa had a concentration of 20 × 10^6^/ml with a total motility of 87% (Supplementary Table SI). However, as with Patient 1, exposure to progesterone elicited no [Ca^2+^]_i_ response or stimulation in the viscous media penetration test (Fig. 2A and E). Additionally, similarly to Patient 1, the spermatozoa were able to significantly hyperactivate when exposed to 2 mM 4-AP (*P* = 0.01) (Fig. 2C). All nine oocytes inseminated by IVF failed to fertilize. Female age was 24 years, with an AMH of 8 pmol/l.

Patient 2 returned for ICSI treatment 7 months later. Three out of five oocytes normally fertilized. Analysis of this sample showed that treatment with progesterone induced both a [Ca^2+^]_i_ signal and a significant response in the viscous media penetration test (data not shown). Therefore, Patient 2 was not recalled for further analysis for electrophysiology.

### Donor 1

Donor 1 was a healthy young male who had never contributed to a pregnancy. Two semen analyses showed an average sperm concentration of 109 × 10^6^/ml with a total motility of 69%. In the initial sample, the sperm were unable to produce a [Ca^2+^]_i_ response to progesterone (Supplementary Fig. S2A) and were not able to respond to progesterone in the viscous media assay (Supplementary Fig. S2D): an outcome similar to that of Patient 1 and the first sample provided by Patient 2. However, the spermatozoa were able to produce a significant HA response when treated with 4-AP (Supplementary Fig. S2B, *P* = 0.0112). Donor 1 produced another sample 1 week after the initial sample. Spermatozoa from the second sample showed a clear [Ca^2+^]_i_ response when stimulated with progesterone (Supplementary Fig. S2A) and significant HA when stimulated with 4-AP (Supplementary Fig. S2C), but failed to respond to progesterone in the viscous media assay (Supplementary Fig. S2E). Therefore, Donor 1 was not recalled for further study for electrophysiology.

### Electrophysiology

**Figure 3:**
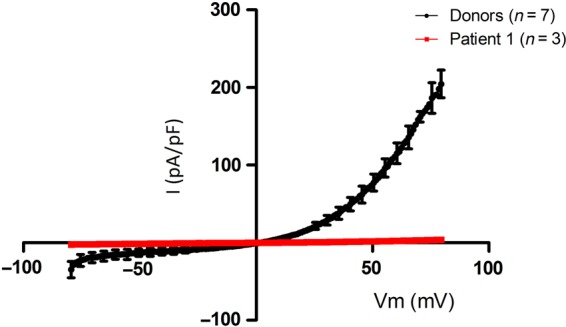
Whole-cell patch clamp recordings from spermatozoa from Donors and Patient 1. CatSper currents are absent from spermatozoa from Patient 1. Current–voltage relationship recorded under Cs-based divalent free conditions from spermatozoa from Patient 1 (*n* = 3). Capacitated donor data are also shown (*n* = 7). Current is measured in picoamps per picofarad, to normalize for variation in capacitance between cells. Error bars represent standard error of the mean (SEM).

CatSper has typically been characterized under monovalent conditions (Lishko *et al.*, 2011; Smith *et al.*, 2013); therefore Cs-based divalent free solutions were used to examine CatSper channel function. CatSper currents (*I*_CatSper_) were robust in sperm from donors with a maximum outward current of 204.6 ± 17.7 pA/pF (Fig. 3). In contrast, *I*_CatSper_ was essentially absent in sperm from Patient 1 with the maximum current (4.0 ± 0.4 pA/pF) being significantly lower (*P* < 0.01).

### Genetic analysis

In mice all seven members of the CatSper Complex (CatSperα(1-4), CatSperβ, γ & δ) are required for functional expression. Assuming a similar requirement in human would suggest that a genetic lesion in any single gene could result in the absence of CatSper conductance. Exome sequence for Patient 1 was analysed for the presence of single nucleotide polymorphism variants and in/dels. This analysis revealed no evidence for loss of function mutations in any of the CatSper Complex loci (Supplementary Table SII). We further analysed a set of genes which, based on mouse genetics, would lead to fertilization failure without affecting spermatogenesis. Again no loss of function mutation was observed (Supplementary Table SII).

## Discussion

In previous studies genetic analysis has identified mutations in the genes coding for proteins of the CatSper channel. In humans, these abnormalities occur in association with other phenotypic abnormalities (Avidan *et al.*, 2003; Avenarius *et al.*, 2009; Hildebrand *et al.*, 2010; Jaiswal *et al.*, 2014). Analysis of CatSper currents has been undertaken in only one of these cases (Smith *et al.*, 2013) where there was a 70 kb deletion which spans four loci, CATSPER2, Stereocilin, CKMT1 and KIAA0377 (Avidan *et al.*, 2003). Deletion of these additional loci may explain the observation that, in addition to failure of CatSper currents, semen from this man was grossly abnormal thus making it unclear if the spermatogenesis defect was due to a loss of CATSPER2 or one of the other 3 loci. In the current study we have screened for functional deficiencies consistent with putative defects of CatSper function in men with normal semen parameters attending for IVF treatment. Our data support the conclusion that loss of CatSper conductance is rare, may occur independently of abnormalities associated with spermatogenesis and, importantly, has functional significance.

The data described here and previously (Alasmari *et al.*, 2013a) show that, on average, sperm from IVF patients responded similarly to donors. There was no significant difference between the groups for (i) Ca^2+^ responses to activation of CatSper or (ii) performance in penetration in the viscous media assay (artificial mucus) under control conditions and when stimulated with progesterone. However, samples from three of the men studied (two IVF patients and one donor) combined normal semen parameters with striking CatSper functional abnormalities. Motility parameters, including HA, were normal (and stable for up to 6 h) as was penetration of the sperm into viscous media in the absence of progesterone. However, these sperm failed to respond to activation of CatSper by progesterone (Lishko *et al.*, 2011; Strünker *et al.*, 2011) either with elevation of [Ca^2+^]_i_ or a functional response in the viscous media penetration test, which is via activation of CatSper (Alasmari *et al.*, 2013b). Samples from two of these men were also used for IVF and both showed complete failure to fertilize. In contrast, only 14/111 patient samples that had a normal progesterone-induced [Ca^2+^]_i_ response (including 9 who previously achieved low/failed fertilization at IVF) failed to fertilize. This is a clear significant difference (*P* < 5*10^−4^; Chi square contingency test) strongly supporting the concept that functional failure of CatSper (progesterone induced [Ca^2+^]_i_) is sufficient to compromise fertility of human sperm, although such defects are rare. Furthermore, the ‘stable’ loss of CatSper function (see below) was not associated with poor semen characteristics. This is in contrast to the mouse phenotype and suggests that the roles of the CatSper complex in normal physiology differ between mouse and human.

Two of the men whose sperm failed to respond to progesterone (Patient 2 and Donor 1) provided further samples (interval of 1 week for Donor 1 and 7 months for Patient 2) that gave [Ca^2+^]_i_ responses upon stimulation with progesterone (e.g. Supplementary Fig. S2c). The assumption is that these men, therefore, have no CatSper genetic lesions. However, sperm from Patient 1 consistently failed to respond to stimulation with progesterone in two samples, and electrophysiological study confirmed the absence of CatSper currents. This suggests the presence of a consistent functional lesion of CatSper in Patient 1 that was aetiologically distinct to that seen in Patient 2 and Donor 1. The most obvious candidate is a genetic alteration. However, no candidate mutations in the CatSper complexes were observed.

Previous analysis of the impact of CatSper loss has been in patients with deletions in multiple loci. This resulted in diverse defects in the spermatozoa raising the question of whether CatSper function is required for normal spermatogenesis (Avidan *et al.*, 2003; Smith *et al.*, 2013). In contrast, our data suggest that spermatogenesis is normal in men where CatSper function cannot be detected in ejaculated spermatozoa. Together with the observation that loss of CatSper activity may be transient (Patient 2 and Donor 1), this further emphasizes the potential of CatSper interruption as a feasible target for contraception.

The absence of any overt genetic abnormality in Patient 1 together with the transient phenotype in Patient 2 and Donor 1 suggests a defect(s) in testicular and/or epididymal sperm maturation and/or potential processing in the mature cell. However, although impaired localization and/or assembly of the CatSper complex is one explanation for loss of function, detailed proteomic studies combined with high quality co-localization and imaging (Chung *et al.*, 2014) would be required to address this.

In this study, and that of Alasmari, the number of men whose partners had IVF and thus had normal semen profiles (concentration and motility) yet defective Ca^2+^ influx induced by progesterone was small (1% and 0%) respectively. Similarly, in the 10 patients with a history of fertilization failure/low fertilization at IVF only one showed a defective [Ca^2+^]_i_ response (Patient 1). Although these findings indicate that CatSper defects are not a common cause of fertilization failure/reduced success, there are caveats to this conclusion. Firstly, since samples from 22% of IVF patients could not be screened (due to practical, technical and/or logistical reasons) the incidence of significant abnormalities might be underestimated. Secondly, the strict definition of a defective Ca^2+^ influx used is such that only substantial abnormal responses were selected for further study. Electrophysiological analysis was performed on Patient 1 with a clear and consistent defect and the sperm from this man showed negligible CatSper current. However, more subtle abnormalities of CatSper function, for example significantly reduced but not absent current, may occur and have some biological significance.

Our studies also shed light on the link between the CatSper complex and the regulation of HA. We demonstrated that 4-AP enhanced HA in sperm from Patient 1, who consistently showed failure of progesterone-induced [Ca^2+^]i elevation and was null for CatSper current. The progesterone-insensitive sample from Patient 2 also responded to 4-AP with an increase in HA. This is consistent with a model for regulation of HA in which the Ca^2+^ stored at the sperm neck is pivotal to HA induction and CatSper-mediated Ca^2+^ influx in the flagellum acts as a trigger for mobilization of this store (Alasmari *et al.*, 2013a,b). Mobilization of stored Ca^2+^ in mouse sperm null for CatSper has also been shown to induce HA (Marquez *et al.*, 2007). Intriguingly, this suggests that pharmacological mobilization of stored Ca^2+^ can bypass failure due to impaired function or regulation of CatSper. Further investigations into the role of calcium response to progesterone and CatSper function, and their relationship to fertilization rate in IVF, should include the use of single-cell Ca^2+^ imaging. This would enable a high resolution investigation into the diverse cellular responses between individuals, and could provide further insight into Ca^2+^ signalling dysfunction.

We conclude that, in IVF patients, a total lack of CatSper current is rare, functionally significant and does not necessarily involve mutation in the CatSper gene complex.

## Supplementary data

Supplementary data are available at http://humrep.oxfordjournals.org/.

## Authors' roles

H.L.W. performed the initial screening on all of the IVF patients, recalling patients and research donors. W.A. performed initial screening and identification of initial recall patients. S.M.d.S., S.M. and H.L.W. were involved in the recruiting, recalling and consenting of patients and donors. S.M. and S.G.B. performed the patch clamping experiments. S.G.B. and S.M. performed the detailed analysis of the electrophysiological data. K.A.S., P.V.L. and M.R.M. were responsible for the genetic analysis. S.M.W., S.J.P., S.G.B. and C.L.R.B. were involved in the design of the study and obtained funding for the experiments. The initial, interim and final manuscript was drafted by C.L.R.B., H.L.W., S.G.B. and S.J.P. All authors contributed to the construction, writing and editing of the manuscript. All authors approved the final manuscript for submission.

## Funding

The initial funding for the project was supported by grants from NHS Tayside, Infertility Research Trust, TENOVUS, Chief Scientist Office NRS Fellowship (S.M.d.S.), the Wellcome Trust, University of Abertay (sabbatical for S.G.B.). P.V.L. was supported by NIH grant R01GM111802 (NIGMS) and Alfred P. Sloan Award. The majority of the data were obtained using funding from a MRC project grant (#4190). Funding to pay the Open Access publication charges for this article was provided by MRC.

## Conflict of interest

None declared.

## Supplementary Material

Supplementary Data
