# Supplementary material for: Specific loss of CatSper function is sufficient to compromise fertilizing capacity of human spermatozoa
Source: Hum Reprod. 2015 Oct 8;30(12):2737–46. doi: 10.1093/humrep/dev243 (PMC4643530; doi:10.1093/humrep/dev243)
Supplement: Supplementary Data [file supp_30_12_2737__index.html]

Specific loss of CatSper function is sufficient to compromise fertilizing capacity of human spermatozoa — Supplementary Data 

# Specific loss of CatSper function is sufficient to compromise fertilizing capacity of human spermatozoa

## Supplementary Data

Supplementary Data

- Supplementary Figure 1 - pdf file
- Supplementary Figure 2 - pdf file
- Supplementary Table 1 - pdf file
- Supplementary Table 2 - pdf file
