# Supplementary material for: Specific loss of CatSper function is sufficient to compromise fertilizing capacity of human spermatozoa
Source: Hum Reprod. 2015 Oct 8;30(12):2737–46. doi: 10.1093/humrep/dev243 (PMC4643530; doi:10.1093/humrep/dev243)
Supplement: Supplementary Data [file supp_dev243_dev243supp_fig1.pdf]

Human Reproduction, 2015
doi:10.1093/humrep/dev243

human reproduction

SUPPLEMENTARY DATA

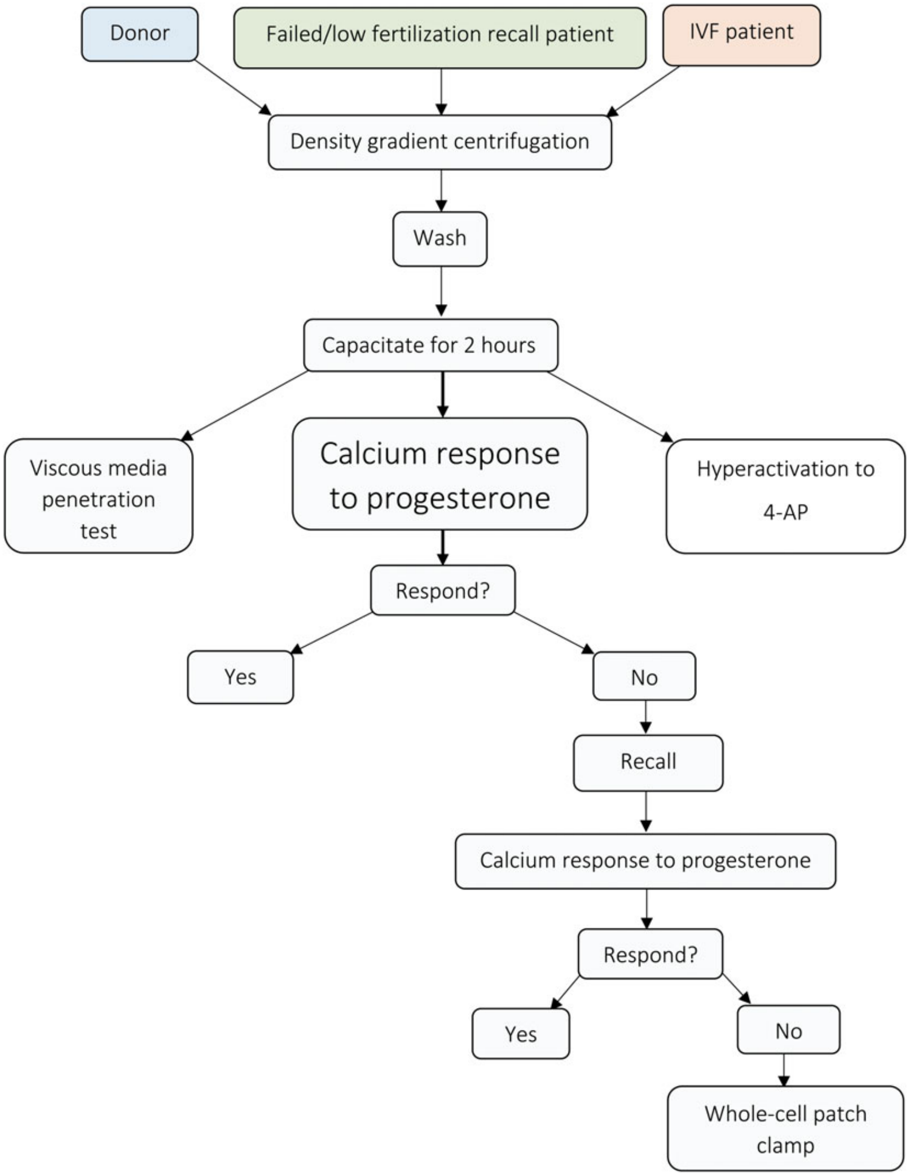

Supplementary Figure S1 Experimental design. Semen from donors, IVF patients and those patients having previous failed/low fertilization at IVF (recall) was prepared using density gradient centrifugation and a wash step. The primary assay was the Ca^{2+} influx induced by progesterone. Depending on cell availability, the samples were then subjected to one or a combination of the progesterone-induced penetration into viscous media; 4-aminopyridine (4-AP)-induced hyperactivation (HA) assay. Spermatozoa from men with a defective Ca^{2+} response were asked to provide additional samples. In men where there was a consistent and robust failure of the Ca^{2+} influx induced by progesterone, i.e. repeatable in more than one semen sample, a direct assessment of CatSper function was performed using electrophysiology (patch clamping).
