# Supplementary material for: Specific loss of CatSper function is sufficient to compromise fertilizing capacity of human spermatozoa
Source: Hum Reprod. 2015 Oct 8;30(12):2737–46. doi: 10.1093/humrep/dev243 (PMC4643530; doi:10.1093/humrep/dev243)
Supplement: Supplementary Data [file supp_dev243_dev243supp_fig2.pdf]

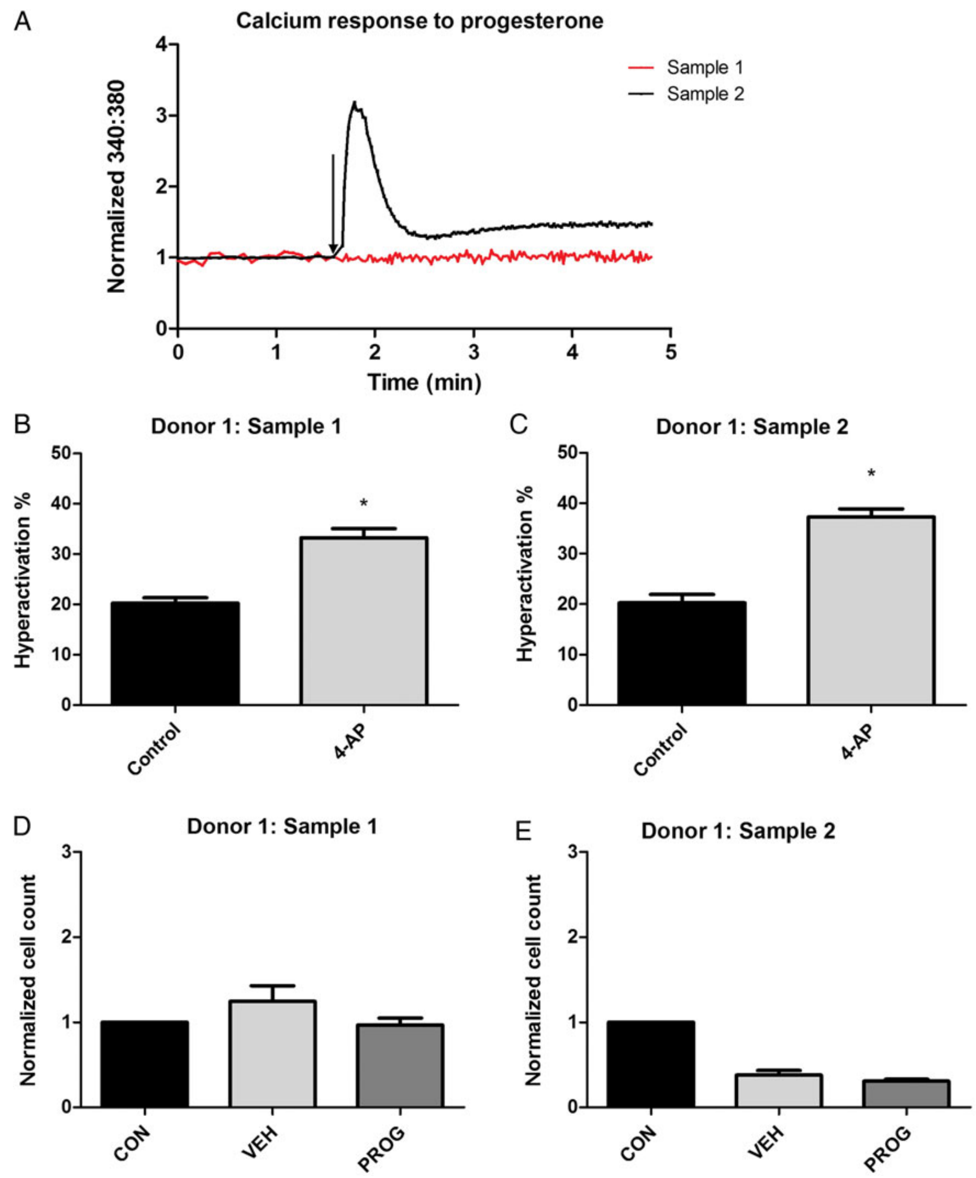

Supplementary Figure S2 Spermatozoa from Donor 1 showed defective calcium response to progesterone, penetration into viscous media. Spermatozoa from the first semen sample produced by Donor 1 were defective in the Ca^{2+} influx induced by progesterone assay (**A**), and similarly showed an absence of response to progesterone in the penetration into a viscous medium (**D**). Hyperactivation (HA) was significantly higher after treatment with 4-aminopyridine (4-AP) (**B**, $P = 0.0112$). Spermatozoa from the second semen sample showed a normal Ca^{2+} influx induced by progesterone (**A**), but penetration into a viscous medium was not enhanced by progesterone E. HA was significantly higher after treatment with 4-AP (**C**, $P < 0.001$).
