# Supplementary material for: Specific loss of CatSper function is sufficient to compromise fertilizing capacity of human spermatozoa
Source: Hum Reprod. 2015 Oct 8;30(12):2737–46. doi: 10.1093/humrep/dev243 (PMC4643530; doi:10.1093/humrep/dev243)
Supplement: Supplementary Data [file supp_dev243_dev243supp_table1.pdf]

Supplementary Table SI **Semen and prepared sperm analysis for Patient 1, Patient 2 and Donor 1.**

	Semen		Prepared sample		
	Count (million/ml)	Motile (%)	Count (million/ml)	Motile (%)	Hyperactivation (%)
Patient 1	66 (54.65)	60 (16.5)	41 (33.85)	92 (5)	11 (2)
Patient 2	80 (2)	55 (2.5)	20 (1.5)	87 (4.5)	28 (1.25)
Donor 1	109 (8.85)	69 (16)	36 (2.4)	91 (1)	22 (1)

Data are mean ($\pm$ SD).
