# Supplementary material for: Specific loss of CatSper function is sufficient to compromise fertilizing capacity of human spermatozoa
Source: Hum Reprod. 2015 Oct 8;30(12):2737–46. doi: 10.1093/humrep/dev243 (PMC4643530; doi:10.1093/humrep/dev243)
Supplement: Supplementary Data [file supp_dev243_dev243supp_table2.pdf]

Supplementary Table SII **Single nucleotide polymorphisms (SNP) identified in candidate genes.**

Gene	SNP ID	Allele change	Amino acid	Minor allele frequency	Coverage	Variant frequency %
CATSPER1	rs1203998	G --> A	G133S	T = 0.3365	20	30
	rs3814747	G --> A	V652I	C = 0.0635	34	100
CATSPER2	rs8042868	G--> A	V63I	T = 0.2035	12	50
	rs550866834	A --> T	V154G	N/A	65	47.7
CATSPER3	None detected					
CATSPER4	None detected					
CATSPERB	rs72629402	T --> C	V248A	G = 0.0561	11	81.8
CATSPERD	rs61180947	G --> A	G639R	A = 0.2296	11	36.4
CATSPERG	rs139891538	C --> T	A5V	T = 0.0004	12	41.7
ATP1A4	rs6427504	G --> A	G83D	G = 0.3069	5	100
	rs17368402	G --> A	E297K	A = 0.0449	42	38.1
ATP2B1-4	None detected					
CLGN	None detected					
IZUMO	None detected					
SLC9C1	rs9809404*	A --> G	I348M	T = 0.3570	41	43.9
	rs9809384*	A --> G	I364V	T = 0.3570	58	32.8
	rs4434123	C --> T	T705I	G = 0.1697	7	100
	rs6781844	C --> A	Q732K	G = 0.1699	7	100
	rs74840030	A--> G	M844V	C = 0.0369	16	43.8
	rs73853324	T --> C	V1058A	G = 0.0531	16	43.8
tACE	None detected					
TPCN1	None detected					
TPCN2	rs2376558	T --> C	L564P	T = 0.2470	18	100
	rs896973	G --> T	V565F	T = 0.3335	10	100
	rs3829241	G --> A	G734E	A = 0.1763	5	100

Exome analysis of Patient 1 revealed no loss of function lesions in any genes of interest studied. Amino acid changes are given for the longest isoform. Asterisks indicate linked SNPs. MAF was derived from 1000 Genomes dataset. CatSper genes of interest are shown first followed by all other genes studied in alphabetical order.
Official Name, Full Name/Alias. CATSPER1-4, cation channel, sperm associated 1-4; CATSPERB, catsper channel auxiliary subunit beta; CATSPERD, catsper channel auxiliary subunit delta; CATSPERG, catsper channel auxiliary subunit gamma; ATP1A4, ATPase, Na+/K+ transporting, alpha 4 polypeptide; ATP2B1-4, ATPase, Ca++ transporting, plasma membrane 1/PMCA1-4; CLGN, Calmegin; SLC9C1, solute carrier family 9, subfamily C (Na+-transporting carboxylic acid decarboxylase), member 1/sNHE1; tACE, angiotensin I converting enzyme (testicular isoform); TPCN1-2, two pore segment channel 1-2, TPC1-2.
